# INCLUSION OF HEARING AND VISION IMPAIRMENTS IN COGNITIVE TRAINING INTERVENTIONS WITH OLDER ADULTS

**DOI:** 10.1093/geroni/igad104.1113

**Published:** 2023-12-21

**Authors:** Francesca Marino, Kening Jiang, Jason Smith, Diefei Chen, Jennifer Deal, George Rebok, Alison Huang

**Affiliations:** Johns Hopkins Bloomberg School of Public Health, Baltimore, Maryland, United States; Johns Hopkins Bloomberg School of Public Health, Baltimore, Maryland, United States; Johns Hopkins Bloomberg School of Public Health, Baltimore, Maryland, United States; Johns Hopkins, Baltimore, Maryland, United States; Johns Hopkins University, Baltimore, Maryland, United States; Johns Hopkins University, Baltimore, Maryland, United States; Johns Hopkins Bloomberg School of Public Health, Baltimore, Maryland, United States

## Abstract

Cognitive training intervention implementation and efficacy should be evaluated among representative samples, including those at highest risk of cognitive decline. Hearing and vision impairments are prevalent among older adults and confer an increased risk of cognitive decline/dementia. Whether cognitive training interventions enroll and are designed to include this important subgroup is unknown. A scoping review of PubMed and PsycINFO was conducted to examine the inclusion of older adults with hearing and/or vision impairment in cognitive training interventions. Eligible articles included English-language cognitive training randomized controlled trials and a study population that was cognitively unimpaired, aged ≥55-years, and community-dwelling. Among the 130 articles in the review, 103(79%) were cognitive training and 27(21%) were multimodal interventions that included cognitive training. Over half the trials systematically excluded participants with hearing and/or vision impairment (n=60, 58%). Few studies measured and reported participant hearing and/or vision impairment (cognitive: n=16, 16%; multimodal: n=3, 11%) or reported incorporating low hearing/vision accessibility features into intervention design (cognitive: n=7, 7%; multimodal: n=0, 0%). Greater representation of older adults with hearing and/or vision impairment in cognitive training interventions is needed and can be achieved by strengthening measurement and reporting of participant hearing and vision, minimizing barriers to participation by increasing accessibility in recruitment and intervention design, and intentional inclusion of older adults with hearing and/or vision impairment. With greater representation of older adults with hearing and/or vision impairment, studies can better evaluate group-specific differences in training effect and begin to tailor interventions to potentially maximize benefit.

